# Assessment of fetal corpus callosum biometry by 3D super-resolution reconstructed T2-weighted magnetic resonance imaging

**DOI:** 10.3389/fneur.2024.1358741

**Published:** 2024-03-26

**Authors:** Samuel Lamon, Priscille de Dumast, Thomas Sanchez, Vincent Dunet, Léo Pomar, Yvan Vial, Mériam Koob, Meritxell Bach Cuadra

**Affiliations:** ^1^Department of Radiology, Lausanne University Hospital and University of Lausanne, Lausanne, Switzerland; ^2^CIBM Center for Biomedical Imaging, Lausanne, Switzerland; ^3^Ultrasound and Fetal Medicine, Department Woman-Mother-Child, Lausanne University Hospital and Lausanne University, Lausanne, Switzerland; ^4^School of Health Sciences (HESAV), University of Applied Sciences and Arts Western Switzerland, Lausanne, Switzerland

**Keywords:** super-resolution reconstruction, magnetic resonance imaging, ultrasound, biometry, fetal brain, corpus callosum, corpus callosum segments, agenesis of the corpus callosum

## Abstract

**Objective:**

To assess the accuracy of corpus callosum (CC) biometry, including sub-segments, using 3D super-resolution fetal brain MRI (SR) compared to 2D or 3D ultrasound (US) and clinical low-resolution T2-weighted MRI (T2WS).

**Method:**

Fetal brain biometry was conducted by two observers on 57 subjects [21–35 weeks of gestational age (GA)], including 11 cases of partial CC agenesis. Measures were performed by a junior observer (obs1) on US, T2WS and SR and by a senior neuroradiologist (obs2) on T2WS and SR. CC biometric regression with GA was established. Statistical analysis assessed agreement within and between modalities and observers.

**Results:**

This study shows robust SR to US concordance across gestation, surpassing T2WS. In obs1, SR aligns with US, except for genu and CC length (CCL), enhancing splenium visibility. In obs2, SR closely corresponds to US, differing in rostrum and CCL. The anterior CC (rostrum and genu) exhibits higher variability. SR’s regression aligns better with literature (US) for CCL, splenium and body than T2WS. SR is the method with the least missing values.

**Conclusion:**

SR yields CC biometry akin to US (excluding anterior CC). Thanks to superior 3D visualization and better through plane spatial resolution, SR allows to perform CC biometry more frequently than T2WS.

## 1 Introduction

The corpus callosum (CC) is the largest brain commissure connecting homologous structures of the cerebral hemispheres, fully developed at 20 weeks of gestation through intricate embryogenesis steps (1). It comprises four sections: rostrum, genu, body, and splenium, arranged from anterior to posterior. Complete or partial agenesis of the CC (cCCA and pCCA) count among the most common prenatal brain malformations, with estimated prevalence ranging from 0.3 to 0.7% in general population and 2% to 3% in neurodevelopmental disorder cases (2). The range of callosal anomalies also includes dysplasias such as hypo- or hyperplasia, where CC is unusually thin or thick. Outcomes of these abnormalities, collectively termed “failed commissuration,” span from normal development to severe delay and are notably influenced by anomaly type and associated malformations (3–6). Beyond diagnosis, predicting neurodevelopmental prognosis remains challenging, as shown by post-natal follow-up studies (7). Thus, assessing fetal CC integrity via precise biometry and morphological analysis is pivotal for prenatal and postnatal management and prognosis evaluation of CC abnormalities (7).

Ultrasound (US) and magnetic resonance imaging (MRI) are complementary tools for assessing fetal brain structural development. In routine 2nd trimester ultrasound, screening for callosal abnormalities is carried out by examining indirect signs on axial views: absent or abnormal cavum septi pellucidi (CSP), ascended third ventricle or colpocephaly. If the screening US reveals indirect signs, the patient is referred to a specialized center where a dedicated neurosonography is performed. This examination includes a mid-sagittal view of the brain and 3D volumes allowing direct visualization of the corpus callosum. If neurosonography confirms agenesis or another anomaly of the corpus callosum, its role is also to specify whether this anomaly is isolated or associated with other fetal brain malformations, such as gyration anomalies. This examination helps to guide genetic investigations and prognosis. It is considered to be the gold standard for detecting fetal cerebral anomalies. Once the CC anomaly is confirmed by neurosonography, fetal brain MRI is also recommended to complete the imaging work-up (8–10). Structural MRI T2-weighted sequences (T2WS) are recommended at around 32 weeks of gestational age (GA), though can also be performed during 2nd trimester, to confirm and characterize or rule out suspected callosal abnormalities and other cerebral malformations (11, 12). Independently from their strengths and weaknesses, these modalities often disagree on CC assessment (3). While US offers better spatial resolution and benefits from 3D reconstruction, fetal position and maternal habitus, among others, can limit its effectiveness. Like 3D-US, MRI provides multiplanar acquisition and also better tissue contrast, but its clinical T2WS acquisitions are sensitive to motion and limited by slice thickness (13). Anatomical inaccuracies in fetal CC shape analysis and measurements can arise due to image orientation errors (14). Postnatally, MRI remains the gold standard for pathological CC analysis, often supporting reclassification of cCCA into pCCA or showing additional brain abnormalities that may change prognosis (1, 12).

Super-resolution (SR) reconstruction of fetal brain MRI addresses the main limitations of T2WS by correcting motion artifacts and enhancing spatial resolution (15–21). By integrating multiple orthogonal scans with thick slices, SR generates motion-free high-resolution images, yielding isotropic 3D MR volumes at 0.5 to 1.2 mm resolutions (22). Multiplanar reconstruction in any plane is pivotal for precise CC measurements, though SR could introduce anatomical distortions (13). Prior studies have explored SR for fetal brain biometry, including whole brain and posterior fossa (23–26), ocular biometry (27) and normative fetal brain atlases (14, 28, 29). However, detailed CC biometry on SR-reconstructed fetal brain MRI remains unexplored. Given the relevance and challenges of prenatal assessment of CC, its validation on MRI against current reference standard (US) is necessary before clinical use. This study evaluate SR’s proximity to US, compared to T2WS, in measuring normal CC and sub-segments while exploring its potential in assessing pCCA.

## 2 Methods

### 2.1 Cohort

**Figure 1:**
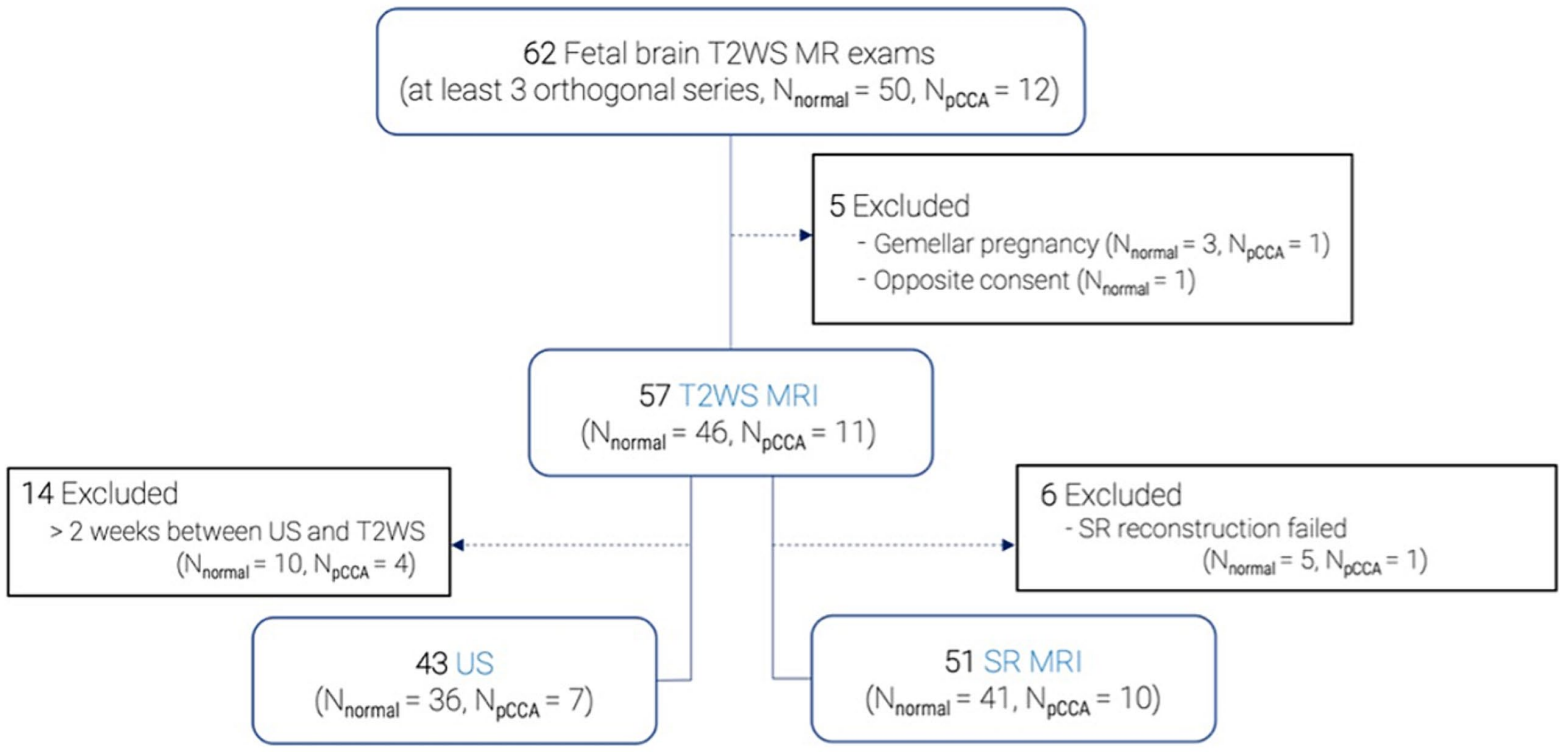
Data selection flow chart.

We retrospectively analyzed fetal brain MRI scans conducted on medical indication at Lausanne University Hospital between 2014 and 2021 (Figure 1). Medical indication was either due to a brain malformation detected on routine ultrasound (for instance partial CCA), because of a risk factor (infection, known genetic abnormality, drug exposure, etc.) or for a history of a brain abnormality in a previous pregnancy. Of 62 selected MRI exams with at least three orthogonal T2WS series, 50 were classified as normal (absence of anomalies or mild ventriculomegaly < 12 mm), while 12 exhibited pCCA, defined by absent or abnormal sub-segment(s). Excluding 4 gemellar pregnancies and 1 rejection of consent, 57 T2WS exams were included: 46 normal (38 without abnormalities, 8 with mild ventriculomegaly) and 11 pCCA. GA, which was determined from last menstrual period and confirmed by early trimester ultrasound, ranged from 21 to 35 weeks in the normal group and 22 to 31 weeks in pCCA.

All images were anonymized for analysis. This retrospective study was part of an approved larger research protocol (CER-VD 2021-00124).

### 2.2 Data

**Figure 2:**
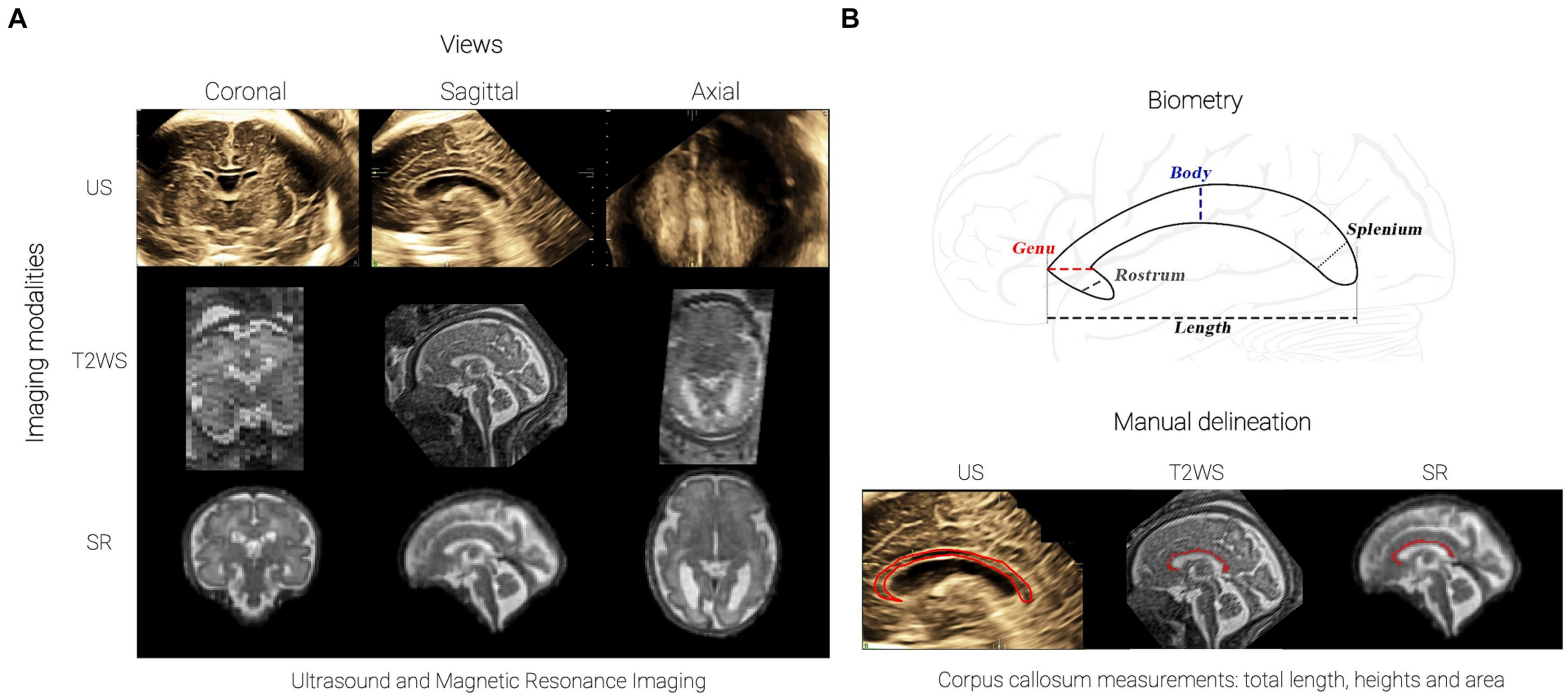
Corpus Callosum Imaging: **(A)** Imaging approaches: 3D ultrasound (top), T2WS MRI (middle, sagittal acquisition reoriented view for clarity; low- resolution T2WS also in coronal and axial orientations), 3D super-resolution (SR) reconstructed MRI (bottom); **(B)** CC and sub-segment biometry (from anterior to posterior: rostrum, genu, body, splenium) and manual CC area delineation (bottom).

Clinical MR images were acquired at either 1.5 or at 3 T (12.5% of exams). There was no specific reason for choosing 1.5 or 3 T at MAGNETOM scanners (Siemens Healthcare, Erlangen, Germany, see details in Supplementary Table S1). The fetal brain MRI protocol included T2-weighted Half-Fourier Acquisition Single-shot Turbo spin Echo (HASTE) sequences in three orthogonal orientations. During T2WS exams, multiple series (also referred to as *stacks*) are obtained: on average, 7 T2WS series were acquired per subject (range of 3 to 20). Figure 2A (middle row) illustrates a T2WS sagittal stack, with good in-plane sagittal resolution but thick slices in coronal and axial views.

These datasets underwent motion correction and 3D super-resolution reconstruction using MIALSRTK pipeline (30), with an isotropic spatial resolution matching its input in-plane resolution (1.1 mm^3^ for 1.5 T and 0.5 mm^3^ for 3 T exams). Example of SR is shown in Figure 2A (bottom row). On average 5.6 T2WS stacks per subject’s reconstruction were used (range of 3 to 9). Six MR exams failed to be reconstructed due to bad T2WS quality. A total of 51 SR exams (41 normal and 10 pCCA) were finally available for measurements.

We selected US exams performed within 2 weeks before or after MR imaging, as recommended by MERIDIAN (31) and various other studies (12, 32) (precise time of acquisition in Supplementary Figure S1). This timeframe balances maintaining a robust framework for comparative biometric analysis while minimizing patient exclusion. This yielded 43 US sessions (36 normal and 7 pCCA) suitable for measurements (Figure 2A, top row). Ultrasounds were performed using General Electric Voluson 730, E8, E10 devices with 5- to 8-MHz 3D transabdominal and transvaginal transducers. Skilled obstetricians or specialized midwives from specialized perinatal ultrasound and fetal medicine unit acquired the images. Among 43 US series, 39 (92.9%) were 3D-US, and only 3 (7.14%) were 2D-US. For 3D-US, brain volumes containing the CC were acquired through optimization from 2D images using bi-parietal diameter or trans-cerebellar axial planes, with resulting 3D volumes being displayed as isotropic multiple orthogonal 2D images (33). Volume contrast imaging (VCI) mode was utilized to generate thin 3D slices, enhancing resolution and contrast, and applied from 1 mm at 18 weeks up to 4 mm at 38 weeks in our study. For 2D-US, images were acquired in mid-sagittal planes also using trans-cerebellar axial view, aligning transducer with anterior fontanelle and sagittal suture as an acoustic window. Measurements of the corpus callosum in 3D have previously been shown to be similar to those obtained in 2D for its length (34).

### 2.3 Measurements

Two observers independently measured CC length (CCL) and sub-segments heights on MRI datasets (T2WS and SR): a junior observer (obs1) with no fetal brain MRI expertise and a senior pediatric neuroradiologist (obs2) with 15 years of experience. Both observers were blinded to clinical data, including GA. Obs1 also measured US images, which underwent multiple reviews and validation, guided by a midwife and an obstetrician, with, respectively, more than 10 and 35 years of experience.

On MR imaging, ITK-SNAP version 3.6.0 (35) was used for biometry and the images were re-oriented to fit in the orthogonal axis. For T2WS, the best low-resolution stack for each orthogonal plane was visually chosen by obs1. On US, measurements were obtained directly on US devices, with navigation to find optimal mid-sagittal plane. All CC measurements were related to the length and height of the hypoechoic area, excluding the boundary hyperechoic structures, and were performed with a 0.1 mm resolution cross-shaped caliper.

All CC measurements (normal and pCCA) were performed in triplicate and conducted on each imaging (US, T2WS, SR) encompassing CCL and heights of rostrum, genu, body and splenium (Figure 2B, top) using established techniques (36):

- Rostrum: postero-inferiorly oriented anterior part.
- Genu: anterior to line through anterior fornix and parallel to line through posterior fornix and quadrigeminal plate.
- Body: between splenium and genu.
- Splenium: posterior 20% of CC.

Manual CC delineation on T2WS and SR used ITK-SNAP’s paintbrush mode (30). CC area was approximated by summing voxels multiplied by pixel size. For US, the CC contour was manually drawn on device, automatically yielding CC area (Figure 2B, bottom). pCCA measurements followed the same methodology as normal cases (see Supplementary Figure S2). A repeatability study using intra-class correlation coefficient was performed remotely on 20% of the dataset (see Supplementary Table S2).

Finally, quality measurements were performed by obs1 across all modalities. T2WS was evaluated (rated as 0 = unusable, 1 = bad, 2 = average, 3 = good) using the mean of 6 items: visualization, whole view and blurring of CC and plane obliquity, motion and blurring of stack. SR was assessed similarly, excluding obliquity and motion, which are irrelevant with SR. US image quality used Pomar et al.’s criteria (34), including sagittal plane, CC parts visibility, sufficient zoom, CC to CSP distinction and proper caliper placement, each criteria rated as yes = 1, no = 0, with final rating of 5 points (good), 3–4 (average), 1–2 (bad) and 0 (unusable).

### 2.4 Regression and statistical analysis

We used normal subject measurements for GA regression. Normative regressions were compared to previously published charts: US vs. Pashaj et al. (36) for all CC biometry and T2WS vs. Tilea et al. for CCL (37). No reference charts for T2WS or SR CC sub-segments measures were found. CC area was compared to US imaging reports (38). Statistical analyses were assessed within measurement subsets: inter-modality agreement (US, T2WS, and SR) for obs1; inter-modality agreement (US vs. T2WS, US vs. SR) between observers, using expert-validated US measures by obs1 and expert-MR (T2WS, SR) measures from obs2. We used paired Wilcoxon rank sum test for each comparison (*p* < 0.05 significance). *p*-values were adjusted for multiple comparisons (5: CCL, rostrum, genu, body, splenium) using Bonferroni correction.

## 3 Results

**Figure 3:**
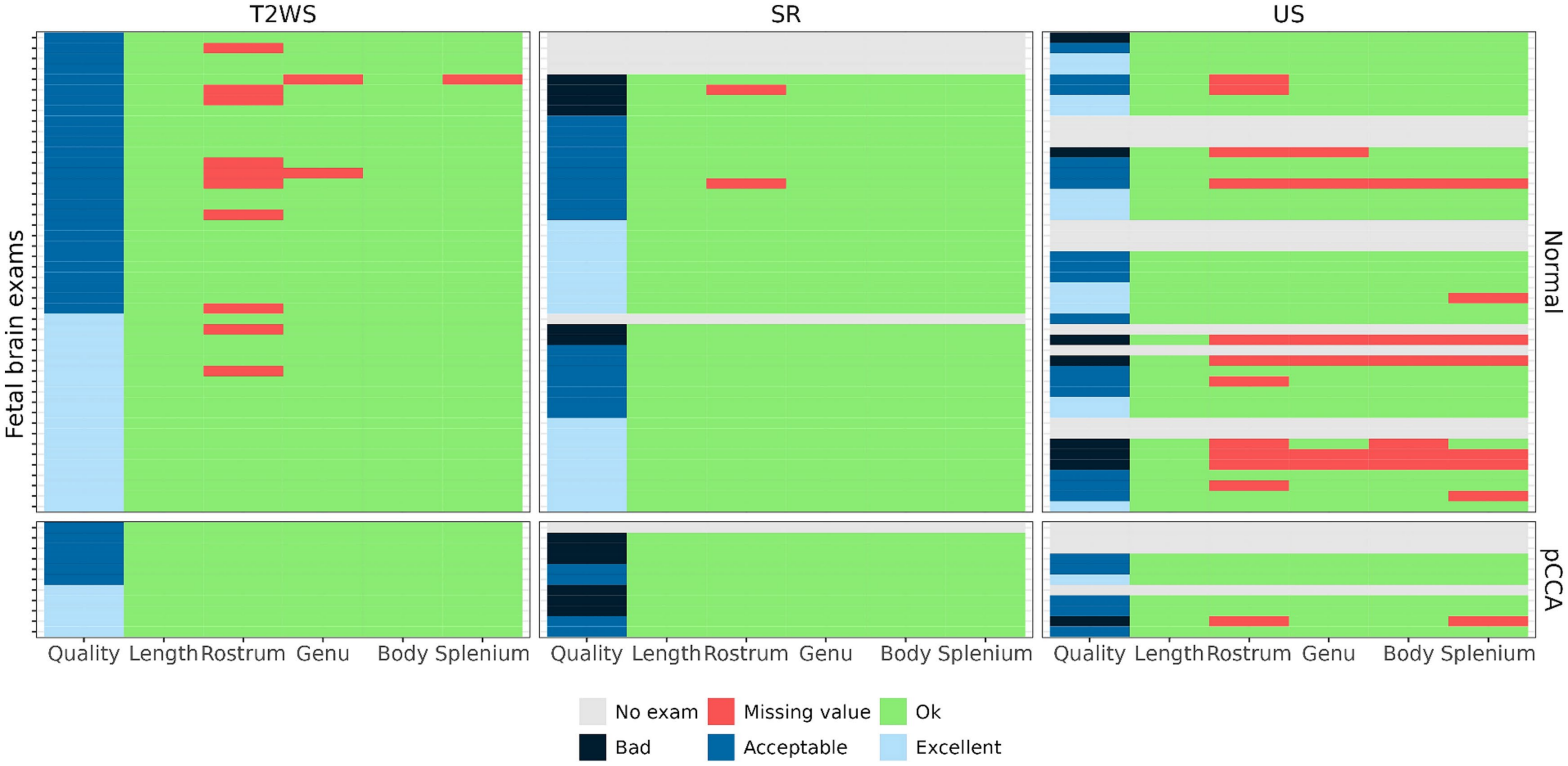
Imaging quality and availability of measurements: CCL was measured 100% in all modalities; the percentage of measurements per imaging method (US/T2WS/SR) are, respectively, 72/82/96 for rostrum, 86/96/100 for genu, 86/100/100 for body and 81/98/100 for splenium.

Figure 3 summarizes quality assessment and missing values across varied imaging methods. The absent values occurred only in CC sub-segments, with a higher incidence in US than in T2WS and SR, spanning all quality levels. Out of 43 US examinations, 14 (32.5%) lacked measurements. For T2WS, among 57 exams, 11 (19.3%) exhibited missing measurements, primarily concerning the rostrum. In SR, merely 2 out of 51 cases (3.9%) featured missing measurements, both linked to the rostrum. However, let us just not forget that 6 SR failed to be reconstructed. If we would count them as missing values, it would raise the percentage to 15.7%. Generally, T2WS MRI’s superior quality to US could be explained, partially, by the initial selection of subjects based on MR exams.

**Figure 4:**
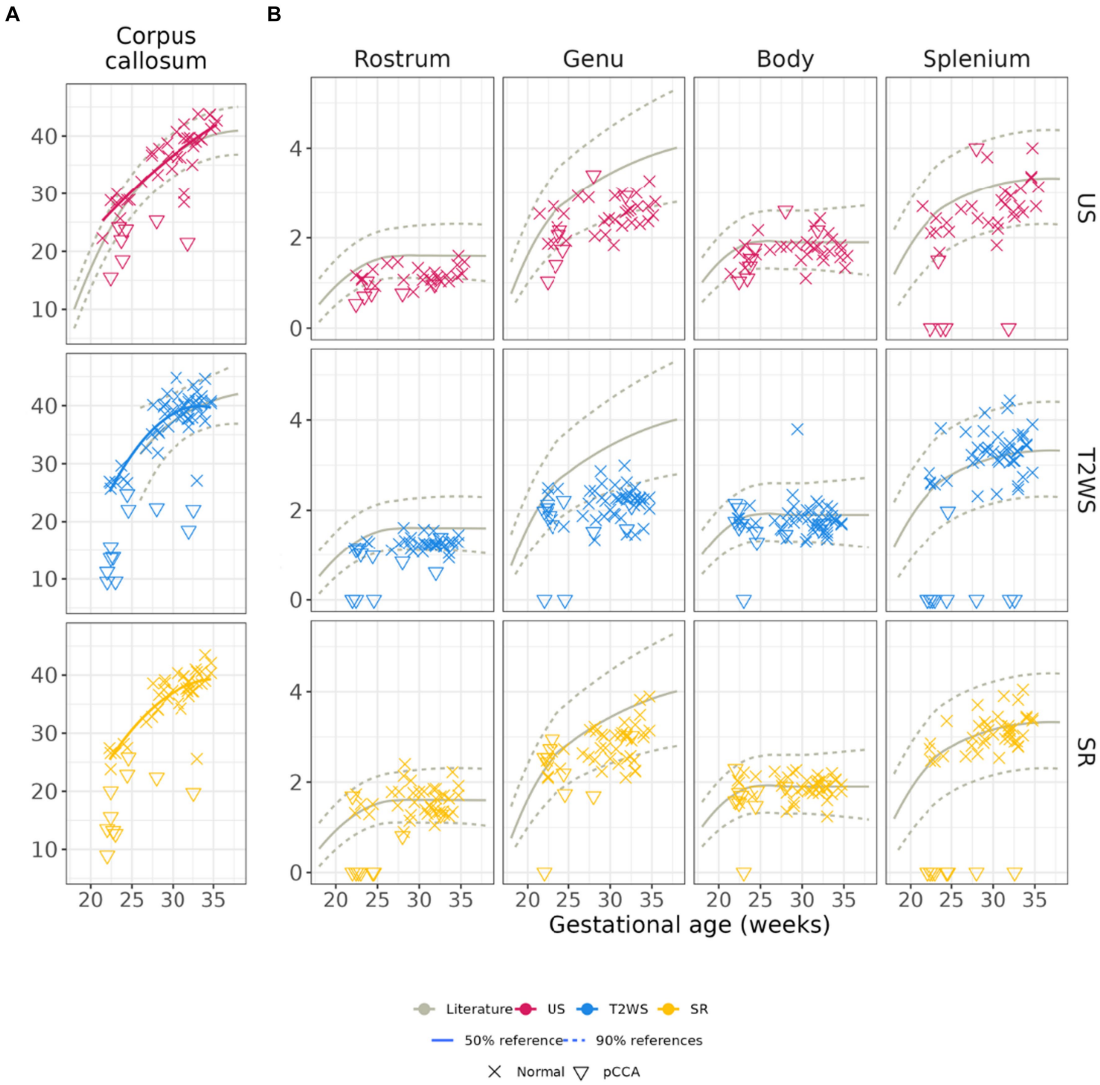
**(A)** Corpus callosum length and heights by obs1. Pathological subjects with partial CCA are shown (illustrated by triangles) but not used for the regression curves. First column: Length of the corpus callosum: **(B)** US and T2WS measurements are, respectively, compared with previous reported values in the literature (gray solid and dashed lines) from US (36) and T2WS MRI (37). Please note that no literature values exist for CCL on SR. Columns 2 to 5: CC heights: previous reported values (36) (solid and dashed lines) from US measurements are overlayed for all three image modalities.

Figure 4A displays intra-obs1’s CCL measurements using US, T2WS and SR. Pathological subjects with pCCA, illustrated by triangles, are not used for regression curves. US and T2WS measurements are compared with reported values from literature (36, 37) showing high agreement. The fourth panel overlays SR regression on US and T2WS measurements. SR measurements fit US better than T2WS.

Figure 4B compares obs1’s CC sub-segment heights measurements in US, T2WS and SR along with literature’s US values (36) only, due to lack of previously reported MR T2WS or SR data. Overall, agreement with previous literature is high for *Body* and *Splenium*, deviating more for *Rostrum* (US) and *Genu* (all imaging). Intra-rater statistical analysis of *CCL* and CC sub-segment heights by obs1 are in Table 1. T2WS and SR show significant differences for *Body, Genu* and *Rostrum*. T2WS and US differ significantly in *Genu* and *Splenium*. SR and US differ in *CCL* and *Genu*.

**Table 1:** Intra-observer (obs1) variability between the different imaging measurements of the CC length and the heights of its sub-segments.

*p*-value (sample size)	CCL	Rostrum	Genu	Body	Splenium
US vs. T2WS	1.0 (*N* = 43)	1.0 (*N* = 24)	5.5e − 03^*^ (N = 35)	1.0 (*N* = 37)	6.7e − 03^*^ (N = 34)
T2WS vs. SR	0.25 (*N* = 51)	2.7e − 02^*^ (*N* = 42)	1.1e − 07^*^ (N = 49)	2.7e − 02^*^ (N = 51)	0.41 (*N* = 50)
US vs. SR	1.4e − 02^*^ (*N* = 38)	0.32 (*N* = 26)	1.7e − 02^*^ (*N* = 32)	0.09 (*N* = 32)	0.14 (*N* = 30)

**Figure 5:**
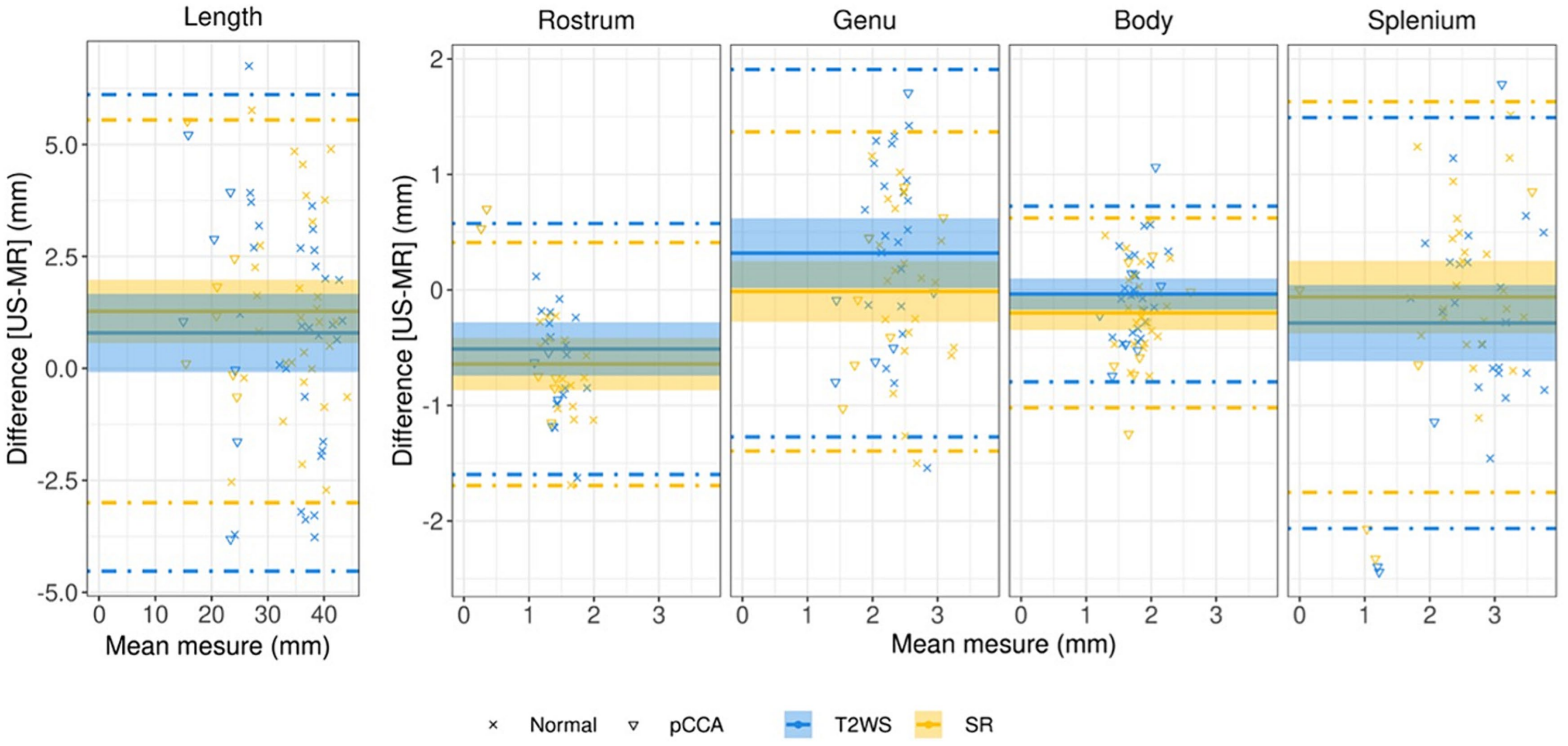
Bland–Altman plots for CCL and CC heights assessing expert US vs. expert MRI measurements (T2WS in blue, SR in yellow) for all normal (cross symbol) and pathological subjects (triangle symbol) with partial CCA. The plot’s x-axis shows the average measurement, while the y-axis shows the difference in measurements between them. The average difference in measurements is represented by the solid line, while the 95 percent confidence interval limits are represented by the dashed lines. Dashed lines = 95% limits of agreement, and shadow areas correspond to the 95% confidence interval (CI).

We analyzed inter-modality performance to mitigate experience effect. Bland–Altman plots in Figure 5 report expert MR measures (obs2) compared to expert US (obs1): negative differences indicate MR relative overestimation; solid lines indicate near 0 average/median differences. Except *Rostrum*, which is approximately −1 mm, few outliers (beyond dashed lines) are present. Additionally, regression of CC measurements with GA, compared with literature is in Supplementary Figures S3, S4. Differences between expert observers are statistically significant for *Rostrum* between US and MR, both for T2WS and SR imaging and *CCL* for US and SR only (Table 2). Lastly, we evaluated inter-observer (obs1 and obs2) biometric measurements within SR, revealing statistical differences in *Genu* and *Rostrum* heights (Table 3).

**Table 2:** Statistical analysis inter-modality by experts US (obs1) and MR (obs2).

*p*-value (Sample size)	CCL	Rostrum	Genu	Body	Splenium
US vs. T2WS	0.42 (*N* = 42)	1.6e − 04^*^ (N = 28)	7.7e − 02 (*N* = 34)	1.0 (*N* = 35)	0.19 (*N* = 34)
US vs. SR	8.2e − 03^*^ (*N* = 38)	9.1e − 05^*^ (*N* = 25)	1.0 (*N* = 31)	6.6e − 02 (*N* = 32)	1.0 (*N* = 30)

**Table 3:** Statistical analysis inter-observers within SR measurements.

*p*-value (sample size)	CCL	Rostrum	Genu	Body	Splenium
SR	1.0 (*N* = 51)	9e − 05^*^ (*N* = 48)	1.1e − 02^*^ (*N* = 50)	8.3e − 02 (*N* = 51)	8.6e-02 (*N* = 51)

**Figure 6:**
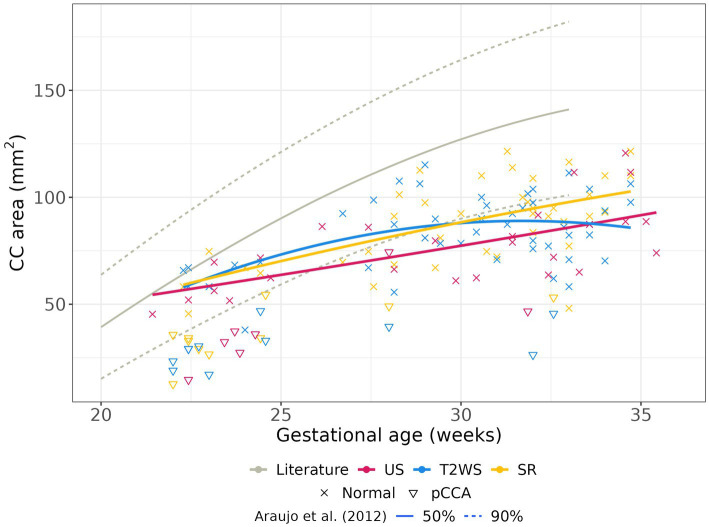
Area of CC with GA: manual delineation on US, T2WS and SR. Pathological subjects with partial CCA are shown (illustrated by triangles) but not used for the regression curves.

Figure 6 displays our CC area estimated across GA. We include literature’s reported results from US (33) in gray. Our US results align with literature for young fetuses (<27 weeks GA), diverging more in later stages (red). Intriguingly, SR (yellow) follows a similar GA trend as US but slightly overestimates the area (same slope). T2WS (blue) exhibits a distinct trend.

## 4 Discussion

### 4.1 CCL can be precisely measured using both MRI methods

Published biometric charts for fetal brain growth via US include *CCL* linear regressions (34, 36, 39, 40). Prior studies also explored fetal brain morphometry using T2WS (37, 41, 42). Two recent studies (25, 26), one from our institution, evaluated *CCL* measurements through SR as compared with T2WS. Our study extends this research, examining *CCL* across GA on T2WS and SR in a larger cohort, reinforcing the reliability of earlier cited works while aligning with expert US measurements. Although CCL measurements between expert US and SR are significantly different in the statistical paired-wise analysis, there is a similar trend on the regression curves according to GA for both SR and US. We hypothesize that this could be due to acquisition time difference between US and MRI, as evoked by the steep slope of *CCL* growth.

### 4.2 New normative CC sub-segments heights measurements on MRI across GA

Historically, fetal CC height assessment relied solely on US due to MRI’s perceived inadequate spatial resolution for small structures (43, 44). Consequently, no normative values were available for CC heights in fetal brain MRI, including T2WS and SR. Our study pioneers CC sub-segments heights analysis and reliability assessment in both MRI methods through linear regressions. SR’s CC heights closely align with US, while both modalities match previous literature (36), except for the anterior CC (*Genu* for obs1, *Rostrum* for obs2) to a lesser extent Observers reported SR’s assistance in *Splenium* evaluation. Enhanced through-plane spatial resolution and 3 orthogonal views likely improve fetal CC visualization in SR. This multiplanar approach reduces confusion risk with adjacent CC structures due to their similar MR image intensities, such as periventricular germ layer, pericallosal and cingulate sulci, Galen’s and internal cerebral veins. Moreover, this error risk intensifies with oblique images, not uncommon in T2WS.

Currently, there is consensus that MR imaging quality of the CC decreases in a postero-anterior pattern. The liquid-rich environment in the posterior CC generates better contrast within brain tissues, aiding CC structure differentiation. As MRI relies on water content while US depends on structure impedance, this explains US superiority in anterior corpus callosum imaging. We postulated that SR holds promise for comprehensive CC visualization. Despite SR’s benefits, our results reveal continued challenges in visualizing the genu on MRI (T2WS and SR) compared to US. SR’s lack of superiority may stem from variable genu size by measurement location, even when anatomically correct. Notably, the genu’s biometry, particularly sensitive to GA, may be affected by the timeframe set between US and MRI acquisitions.

### 4.3 Reduced missing CC sub-segments measurements in SR

Fetal brain MRI biometry can complement US measurements, especially when maternal conditions or fetal position hinder complete US assessment (11). In our study, SR exhibited a lower rate of non-visualized CC segments compared to US and T2WS. SR showed only 3.9% missing values, contrasting with 32.5% in US and 19.3% in T2WS. This has to be balanced with the times where SR cannot be reconstructed. Mainly attributed to poor-quality exams, these omissions concerned *Rostrum*. While US is a favored height measurement method, it’s user-dependent and structure-reliant. SR’s advantage lies in its 3D volume provision, overcoming these limitations. The decreased missing values in SR vs. T2WS may also reflect higher confidence in SR’s CC identification, as suggested in our prior study (25).

### 4.4 Inter-observer variability

Inter-experts analysis reveals that, aside from *CCL* (explained in Section 4.1), *Rostrum* is the singular discrepancy between expert US and expert SR, aligning with Garel et al.’s prior findings (44): the rostrum’s challenge arises due to thinner interhemispheric fissure and lack of cerebro-spinal fluid. It is the thinnest CC part, measuring 1–2 mm (41), akin to SR’s spatial resolution of around 1 mm^3^. This parallels the partial volume effect (13), arising when a voxel contains multiple tissue types, affecting voxel intensity proportionally. Yet, within SR, observer variability indicates disagreement in *Genu* and *Rostrum* heights. In summary, this study provides evidence that measuring CCL, middle and posterior CC sub-segment heights via SR can hold clinical value, despite not being routine. While further validation is needed, it is conceivable that, in situations lacking optimal US but with quality MRI, SR can serve to measure heights relevant to conditions like CC hypo/hyperplasias or dysgenesis (43).

### 4.5 Perspective of CC area

Limited studies have explored CC area and surface, including sub-segments (38, 40, 45). Our gestational CC area findings partly align with Araujo et al. (38), especially for younger fetuses. Notably, SR’s CC area regression aligns with US curve but slightly overestimates it (this could stem from MR’s greater partial volume effects and coarser spatial resolution than US), while T2WS shows a parabolic trend. Abnormally developed fetuses have reported smaller CC areas (46, 47), potentially serving as a biomarker for neurological outcomes. Reliable area measurements, like those from SR, hold increasing diagnostic importance. This significance extends to CC dysplasia, e.g., thick/thin corpus callosum, challenging to diagnose conventionally.

### 4.6 Feasibility in partial CC agenesis

We explored measuring feasibility in pCCA patients (11/57 fetuses). Because of its bidirectional embryogenesis (48), when a default occurs during its period of formation and cause pCCA, the most frequently missing sub-segments will be the most anterior and/or most posterior, respectively rostrum or splenium. Precise CC sub-segments measurements in suspected pCCA is crucial for detecting specific missing parts. While pCCA cases contribute to our study, their small size jeopardizes sub-analysis. pCCA measurements remain on regressions, appearing as clear *CCL* outliers with smaller sub-segments measures. Future research must focus on this population. Connecting prenatal biometry with post-natal neurodevelopment data could aid prenatal and postnatal counseling and management (49–51).

### 4.7 Limitations and extended scope

Our study, although consistent with large-scale US [(36) with 466 samples] and T2WS [(37) with 589 fetuses] investigations, features a relatively small cohort. Retrospective nature and lack of clinical/genetic follow-up (including early-life neurodevelopment and post-natal MRI) are additional limitations shared with fetal brain biometry studies on US (40) and MRI (41). However, following methodological improvements recommended in Rosenbloom et al. (40), we constructed numerical regression charts, maintained age blinding, reported inter- and intra-observer agreement and non-visualization rates. A two-weeks interval between US and MRI imaging could be considered a drawback. However, this timeframe aligns with literature [MERIDIAN cohort (31)] and our study demonstrated an average time difference of 3.9 days (range: 0 to 12 days) between acquisitions. Interpretation of missing values in US images must consider that non-pathological screening brain ultrasounds may lack targeted CC images. Gender impact was not assessed, as sexual dimorphism’s effect on fetal CC remains uncertain, with studies showing both influence (28) and lack thereof (52).

Our focus on SR for CC measurements necessitates future strides toward automating existing SR pipelines and reducing computational time for clinical integration. The potential of SR to enhance diagnosis, therapy and neurological understanding (53) drives our endeavor. Presently, spatial resolution and partial volume effects in MRI limit anterior CC measurements. Thickening millimetric SR images akin to routine CT and MRI practices, or experimenting with finer spatial resolution SR reconstruction, could mitigate these limitations. Investigating variability across SR pipelines is also worthwhile. Furthermore, the comprehensive evaluation of automated segmentation and measurements of CC in normal and pathological fetal brain MRI is still needed. This would allow a more standardized analysis of larger cohorts removing variability in manual measurements.

The endorsement of 3 T fetal MRI acquisitions by the International Society of Ultrasound in Obstetrics and Gynecology (ISUOG) (10) encourages exploration of SR’s value in CC assessment on 3 T images (54). In our study, only 12.5% of cases were imaged at 3 T. We made the choice to pool them with all the 1.5 T cases as a specific sub-analysis in such small sample size would not have allowed to draw conclusions. However, around 30% of fetal exams are nowadays conducted at 3 T, and future research should expand this assessment to a broader multi-centric cohort involving normal and pCCA cases.

Our study is based on ultrasound and MRI screening procedures available in our hospital. Further studies are needed to quantify how different screening procedures (e.g., in middle- or low-income countries) could impact the comparison of US and MRI measurements. Furthermore, more efforts are needed to extend CC analysis to low field MRI scanners, which might be a promising step toward making MRI globally more accessible.

The addition of other imaging modalities and biomarkers would certainly support CC assessment. Incorporating diffusion MRI (55) and MR spectroscopy (46), along with gyrification and neuropathological data (43), as well as long-term neurodevelopmental follow-up, might refine our understanding of CC formation and neurological outlook. Such an approach may lead to more precise classification of CC abnormalities, ultimately influencing prenatal counseling and patient management. Additionally, examining other forebrain commissures could provide further diagnostic clarity, distinguishing commissural defects like pCCA and other forebrain commissures.

## 5 Conclusion

Our study explores the ability of SR fetal brain MRI to depict CC biometry and compare it to US gold standard. Our results show that measurements on SR MRI closely paralleling US except for the anterior segments of the CC. In contrast to US and T2WS, SR consistently enables, if successfully reconstructed, comprehensive biometric measurements, encouraging the adoption of SR for CCL, middle and posterior heights and surface area measurements—especially when US is inadequate in suspected CC anomaly cases. SR fetal brain MRI could be a turning point, combined with other advanced neuroimaging techniques, for a new classification of CC disruptions that could, alongside with genetic and tractography advances, allow a better evaluation of the neurological prognosis, counseling and therapy.

## Data availability statement

The datasets presented in this article are not readily available because of ethical and privacy restrictions. Requests to access the datasets should be directed to the corresponding author.

## Ethics statement

The studies involving human participants were reviewed and approved by the Commission cantonale d’Ethique de la Recherche sur l’être humain du canton de Vaud (CER-VD 2021-00124). The studies were conducted in accordance with the local legislation and institutional requirements. Written informed consent for participation in this study was provided by the participants’ legal guardians/next of kin.

## Author contributions

SL: Data curation, Formal analysis, Investigation, Methodology, Validation, Visualization, Writing – original draft, Writing – review & editing. PD: Data curation, Formal analysis, Investigation, Methodology, Software, Validation, Visualization, Writing – original draft, Writing – review & editing. TS: Data curation, Validation, Visualization, Writing – original draft, Writing – review & editing. VD: Writing – original draft, Writing – review & editing¸ Conceptualization, Data curation, Methodology, Validation. LP: Conceptualization, Data curation, Formal analysis, Investigation, Software, Supervision, Writing – original draft, Writing – review & editing. YV: Conceptualization, Data curation, Resources, Supervision, Validation, Writing – original draft, Writing – review & editing. MK: Conceptualization, Data curation, Formal analysis, Investigation, Methodology, Resources, Software, Supervision, Validation, Visualization, Writing – original draft, Writing – review & editing. MB: Conceptualization, Data curation, Formal analysis, Funding acquisition, Investigation, Methodology, Project administration, Resources, Software, Supervision, Validation, Visualization, Writing – original draft, Writing – review & editing.
