# Supplementary material for: Assessment of fetal corpus callosum biometry by 3D super-resolution reconstructed T2-weighted magnetic resonance imaging
Source: Front Neurol. 2024 Mar 26;15:1358741. doi: 10.3389/fneur.2024.1358741 (PMC11002102; doi:10.3389/fneur.2024.1358741)
Supplement: Supplementary file 1 [file Data_Sheet_1.pdf]

SUPPORTING INFORMATION TO "Assessment of fetal corpus callosum biometry by 3D super-resolution reconstructed T2-weighted MRI"

Samuel Lamon[1,2,3], Priscille de Dumast[1,2], Thomas Sanchez[2,1], Vincent Dunet[1], Léo Pomar[3,4], Yvan Vial[3], Mériam Koob[1,§], Meritxell Bach Cuadra[2,1,*,§]

[1]Department of Radiology, Lausanne University Hospital and University of Lausanne, Lausanne, Switzerland
[2]CIBM Center for Biomedical Imaging, Lausanne, Switzerland
[3]Ultrasound and Fetal Medicine, Department Woman-Mother-Child, Lausanne University Hospital and Lausanne University, Lausanne, Switzerland
[4]School of Health Sciences (HESAV), University of Applied Sciences and Arts Western Switzerland, Lausanne, Switzerland

§ Mériam Koob and Meritxell Bach Cuadra should be considered joint senior author

***Corresponding author**: **Meritxell Bach Cuadra**, Radiology Department, PET3/02/210, Rue du Bugnon 46, CH-1011 Lausanne, Switzerland, +41213146775, meritxell.bachcuadra@unil.ch*

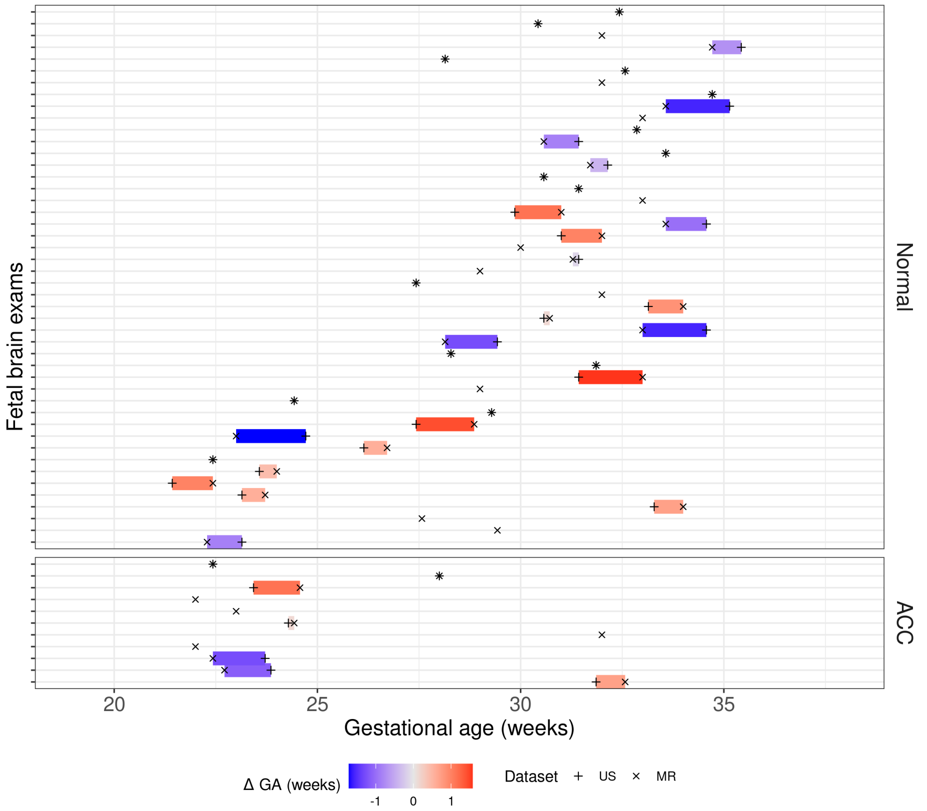

Figure S1 Gestational age at moment of Ultrasound (+) and MR (x) acquisition. Dark colors (blue or red) illustrate the largest time difference.

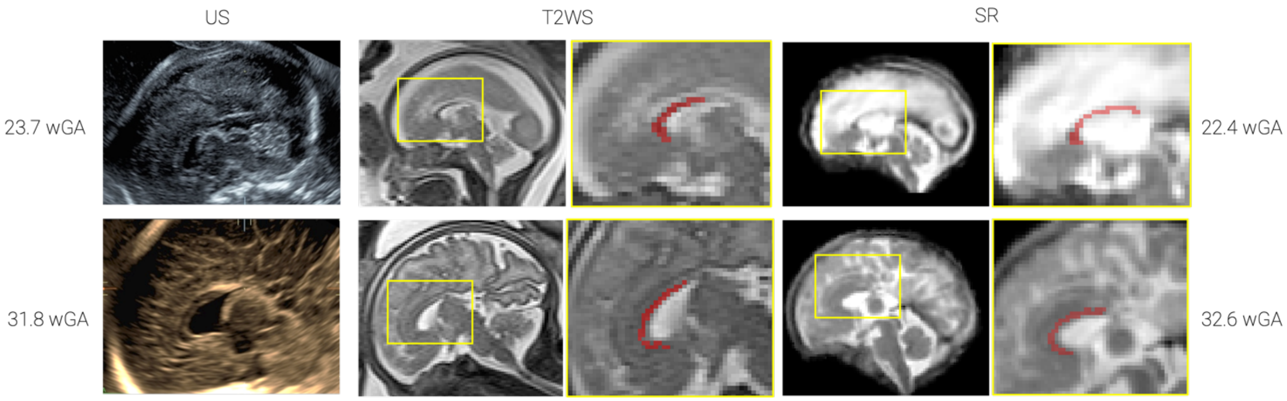

Figure S2 Examples of pCCA with the three imaging methods: patient (top row) is around 23 weeks of GA (23.7 wGA at time of US, and 22.4 wGA at time of MRI); patient (bottom row) is around 32 weeks of GA (31.8 wGA at time of US and 32.6 wGA at time of MRI).

Table S1: Imaging parameters for the subjects in the study. The MRI models are various Siemens MAGNETOM scanners: field strength (T), number of subjects (normal,pathological), TR (ms), TE (ms), original image resolution (mm x mm x mm), field of view (mm), number of stacks used for super-resolution reconstruction (average $\pm$ standard deviation).

**MRI model**	Field strength [T]	Number of subjects (n_{ctrl} n_{patho})	TR [ms]	TE [ms]	Original image resolution [mm^3]	FoV [mm]	Number of stacks for SRR
Aera	1.5	(23, 7)	1200	90	1.1x1.1x3.3	35.8	$5.8_{\pm 2.0}$
Sola	1.5	(18, 2)	1200	90	1.1x1.1x3	35.8	$5.9_{\pm 1.4}$
Skyra_fit	3	(2, 1)	1100	101	0.55x0.55x3	35.2	$6.3_{\pm 0.9}$
Skyra	3	(1, 1)	1100	101	0.55x0.55x3	35.2	$6.0_{\pm 1.0}$
Vida	3	(2, 0)	1100	101	0.55x0.55x3	35.2	$6.5_{\pm 0.5}$

Table S2 Intra class correlation coefficient (ICC) for observer 1. Average on 9 normal and 3 pCCA repeated measurements.

**ICC (Obs1)**	**US**	**T2WS**	**SR**
**LCC**	0.999	0.996	0.995
**Splenium**	0.979	0.993	0.979
**Body**	0.832	0.707	0.145
**Genu**	0.821	0.812	0.536
**Rostrum**	0.887	0.903	0.579
**Overall**	1	0.99	0.99

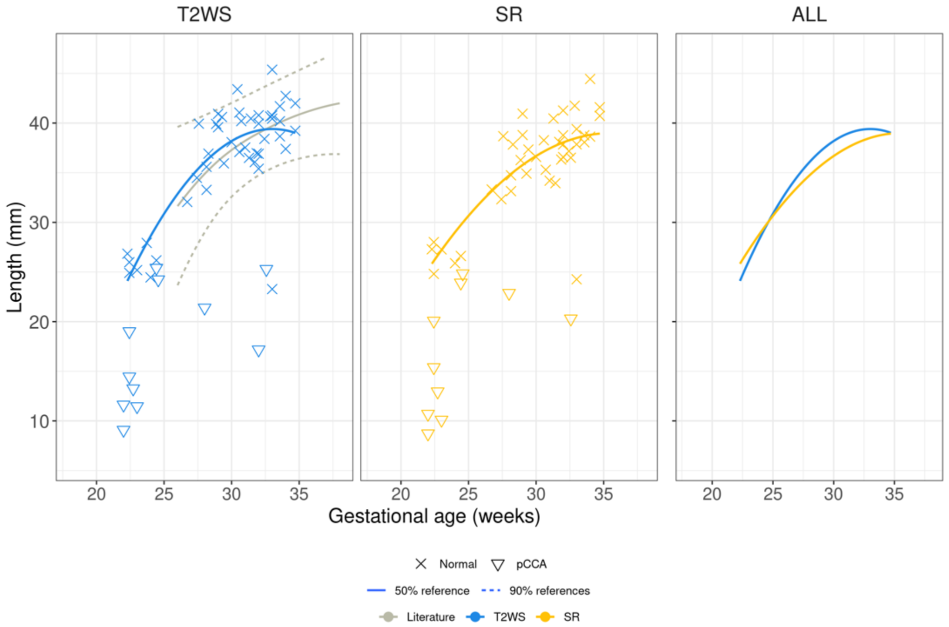

Figure S3 Regression of **LCC** measurements of Obs2 (expert MR) with gestational age.

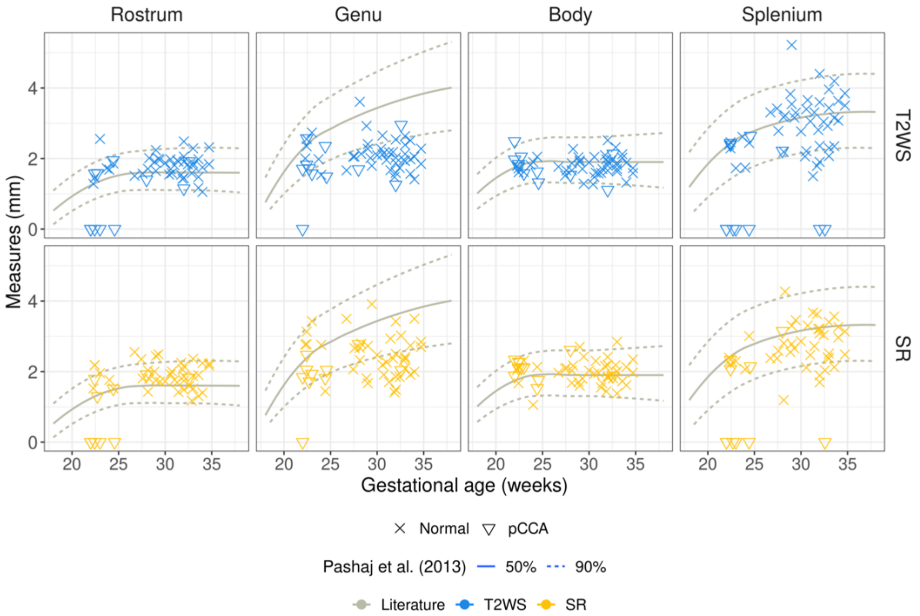

Figure S4 Regression of CC heights measurements (Rostrum, Genu, Body and Splenium) of Obs2 (expert MR) with gestational age.
